# Metabolomics of Male Infertility: A New Tool for Diagnostic Tests

**Published:** 2019

**Authors:** Bahareh Mehrparavar, Arash Minai-Tehrani, Babak Arjmand, Kambiz Gilany

**Affiliations:** 1- Reproductive Biotechnology Research Center, Avicenna Research Institute, ACECR, Tehran, Iran; 2- Nanobiotechnology Research Center, Avicenna Research Institute, ACECR, Tehran, Iran; 3- Cell Therapy and Regenerative Medicine Research Center, Endocrinology and Metabolism Molecular Cellular Sciences Institute, Tehran University of Medical Sciences, Tehran, Iran; 4- Integrative Oncology Department, Breast Cancer Research Center, Motamed Cancer Institute, ACECR, Tehran, Iran

**Keywords:** Biomarkers, Human seminal plasma, Male infertility, Metabolomics

## Abstract

Infertility is a major health issue worldwide. Males and females contribute equally to this problem. Diagnostic semen analysis fails to identify 50% of male infertility disorders. In this regard, metabolomics as a new field of omics has been suggested to have the potential of solving and diagnosis of the male infertility problems. Metabo-lome has a history of around 20 years. However, there are only limited metabolomics studies carried out regarding male infertility. In this review, the current metabolomics researches that have been done in infertile men were reviewed. Based on our own results, using human seminal plasma for metabolomics studies is highly recommended to find potential biomarkers and developing diagnosis tests for detection of main deficiencies in infertile men.

## Introduction

Infertility affects 15% of couples and male factor is associated with almost half of them. About 50% of male infertility etiologies are unknown. In spite of so many studies on male infertility treatments and diagnosis, there are a lot of questions that need to be addressed (1–3). Male infertility is regarded to be a complex disease with its root in genomic, transcriptomic, proteomic and metabolomic areas. The origins of male infertility are not well known, such as asthenozoospermia teratozoospermia, or non-obstructive azoospermia. Furthermore, there are patients with normal semen parameters which are called idiopathic infertile men. The techniques which are used to diagnose the male infertility are mainly macroscopic tests rather than microscopic ones (4). Bio-markers (Biological markers) have different definitions. Broadly, biomarkers are defined as a biological measure of a biological state. In particular, a mutual agreement between the International Program on Chemical Safety, led by the World Health Organization (WHO) in coordination with the United Nations and the International Labor Organization, has defined a biomarker as “any substance, structure, or process that can be measured in the body or its products and assists to predict the incidence probability of outcome or disease” (5).

The recent advances in the use of high-throughput techniques such as genomics, transcriptomics, proteomics and metabolomics give hope to infertile men for a better diagnosis and treatment. State-of-the-art metabolomics technology is becoming a key tool to understand and diagnose the male infertility. Metabolomics as a latest high-throughput technology promises rapid development toward real applications in biomarker identification and diagnosis (3). Searching the Google scholar, PubMed and Scopus databases by combining the terms metabolomics and male infertility apparently shows a few related studies. Almost 15 documents can be found in these databases. In this review, the related metabolomics definitions and the handful of studies that have been done in male infertility have been outlined.

### Metabolomics associated definition:

Metabolomics is the study of cells, tissues or biological fluids by evaluating their metabolites as many as possible. Metabolites are the final products of cellular processes and can be considered as the last reactions to biological arrangements of ecological alterations. The sequences of metabolites created by a biological organization comprise their “metabolome” (6, 7).

The assessment of metabolite level according to its regulatory outcomes as a part of biochemical pathway and based on its application as a diagnostic marker for a variety of diseases has been reported to be crucial. Over the past 20 years, since the first term of the metabolome has been defined, different terms associated with metabolite quantification and quality measurement have been further recommended that include metabolome mapping, metabolic fingerprinting, metabolic profiling, metabolic footprinting, metabolic target analysis to untargeted metabolic profiling, metabonomics and exometabolomics (3, 8).

The typical instruments applied in the metabolomics studies are optical and non-optical spectroscopies including Raman spectroscopy, liquid-chromatography mass spectrometry (LC-MS), gas-chromatography mass-spectrometry (GC-MS), and nuclear magnetic resonance (NMR) spectroscopy. The instrument selection is directly dependent on the type of the metabolomics study (3).

### Metabolomic biomarkers and male infertility:

In the current review, an attempt was made to solely focus on the metabolomics study involving the male infertility. All the articles from PubMed, Scopus and Google scholar databases were retrieved by searching the keywords: “metabolomics”, “metabonomics”, “metabolomic profiling”, “metabolomics fingerprinting” and “metabolome” combining with male infertility.

Human seminal plasma is considered as an important source to study male infertility for a long period of time. One of the earliest studies that used human seminal plasma for the identification of biomarker by metabolomics profiling approaches was done by Hamamah et al. using NMR technology. They analyzed four different groups of spermatogenic failure subjects, vasectomised, very severe oligoasthenozoospermia (OAT) and normozoospermic subjects (Control). They clearly demonstrated the changes of glycerylphosphorylcholine, citrate and lactate levels in azoospermia group compared to normal. Additionally, the ratio of citrate: lactate and GPC: lactate was meaningfully different between the control group and spermatogenic failure or vasectomized groups based on their study (9).

An easy method to analyze human seminal plasma is the metabolomics fingerprinting. Our team apparently showed the efficiency of metabolic fingerprinting for detection of azoospermia changes at the metabolome level (10).

Da Silva et al. performed an interesting study using the metabolomics profiling of human seminal plasma lipids from the spinal cord injury in infertile patients. They found the lipids that mainly belong to the glycerolipids, glycerophospholipids, prenol lipids, fatty acyls and polyketides are deregulated in these patients (11).

Jayaraman et al. used the metabolomics profiling of human seminal plasma to analyze the different groups of infertile men compared to fertile with proven fertility. The infertile groups in their study were idiopathic infertile, oligozoospermia, asthenozoospermia, azoospermia and teratozoospermia. They were able to show different metabolite bio-markers that were up-regulated or down-regulated such as arginine, lysine, tyrosine, citrate, fructose and proline (12). Using metabolomics fingerprinting approaches of human seminal plasma, our group clearly showed the significant changes at the metabolome level in the asthenozoospermia men (13). In the most recent study, using a non-targeted metabolomics profiling approach based on NMR spectroscopy, human seminal plasma of asthenozoospermia men was investigated. Zhang et al. found 19 metabolites that were deregulated in the asthenozoospermia infertile men. The identified metabolites were involved in up-regulation or down-regulation of some amino acids, changes in lipids metabolism, cholesterol metabolism, phospholipids metabolism, the Krebs cycle, nucleoside metabolism and energy metabolism. The most up-regulated metabolites belonged to oxysterols such as 5α-cholesterol and 7-ketocholesterol in human seminal plasma of asthenozoospermia. These metabolites clearly indicated an imbalance of oxidative stress in asthenozoospermia (14).

In order to get better understanding of Kidney-Yang deficiency syndrome (KYDS), seminal plasma of infertile males with KYDS was analyzed by an untargeted metabolomics profiling approach. Chen et al. found 41 deregulated metabolites as potential biomarkers. Seven metabolites were associated to five potential metabolic pathways related to the biosynthesis and metabolism of aromatic amino acids, TCA, sphingolipid metabolism. They suggested the metabolites changes may explain the etiology of KYDS-associated with male infertility (15).

The non-targeted metabolomics profiling approach was used to develop a noninvasive diagnostic technique for detection of spermatogenesis using human seminal plasma of non-obstructive azoospermia patients. In our previous study, 36 metabolites were identified as potential bio-markers. Furthermore, the result shows the testicular sperm extraction (TESE) negative patients have very heterogeneous pattern compared to TESE positive and fertile men (16). Additionally, by applying metabolic fingerprinting, it was shown that this technique can be developed as a fast diagnostic tool for detection of spermatogenesis from non-obstructive azoospermia using seminal plasma as a biological material (17).

Most recently, Zhang et al. performed a metabolomic profiling of serum in non-obstructive azoospermia (NOA) patients. They identified 24 potential metabolites involved in cell apoptosis, oxidative stress and energy production. The potential identified metabolites showed several disrupted metabolism pathways in the NOA patients (18).

Oxidative imbalance has been one of the first observations in the infertile men. It is postulated that the imbalance between reactive oxygen species (ROS) and antioxidant can lead to damage of the human spermatozoa which causes male infertility. One of the earliest metabolomics study on male infertility was based on oxidative stress. An increased level of ROS has been observed in infertile men (19, 20). It was shown that the idiopathic/unexplained infertile men have higher levels of ROS compared to normal fertile men as determined by metabolomics fingerprinting (21). Therefore, introducing a new parameter for semen analysis based on metabolomics has its potential merits.

Moreover, ROS level was measured using meta-bolomics fingerprinting in the non-obstructive azoospermia TESE positive and TESE negative cases. It was demonstrated that the ROS level was extremely higher in TESE negative compared to TESE positive (17)

Shanlei Qiao et al. performed a metabolomics profiling of unexplained male infertility (UMI) based on GC-MS. They found a variety of amino acids including Tryptophan, Serine, Valine, Phenylalanine, Glycine, Proline, Isoleucine and Threonine which were down-regulated in seminal plasma of UMI. In contrast, they have demonstrated the increased levels of urea and Glutamine in UMI seminal plasma. They also asserted 4-Hydroxyphenylacetic acid as an antioxidant was a key metabolite in differentiating the UMI and controls (22).

### Urinary metabolomics and male infertility:

Recently, Zhang et al. did two metabolomics profiling studies using urinary metabolome as a biological source to find potential biomarkers in infertile men. They analyzed the urinary of two groups of infertile men: I) oligozoospermic men; II) normospermic/idiopathic infertile men. In the first study, they identified 10 metabolites as potential biomarkers to diagnose infertility in oligozoospermic men. The down-regulated metabolites of acylcarnitines, aspartic acid, leucylproline and the up-regulated metabolites of adenine and methylxanthine showed a strong association with oligozoospermic risk (23).

In the second study, the urinary of idiopathic infertile men was used to identify 37 metabolites as potential biomarkers which played a major role in energy management, antioxidation and hormone organizing in spermatogenesis. They suggested combining the biomarker pattern of leukotriene E4, 3-hydroxypalmitoylcarnitine, aspartate, xanthosine and methoxytryptophan for diagnosis of deficiencies caused infertility in normospermic/ idiopathic infertile men (24).

### Plasma metabolomics and male infertility:

Recently, plasma metabolome has been subjected to the metabolomics profiling study of infertile men. Zhou et al. analyzed the plasma metabolome of the erectile dysfunction (ED) and semen abnormalities (SA) in infertile men. They identified 1,5-Anhydro-sorbitol and α-hydroxyisovaleric acid metabolites as potential biomarkers to distinguish fertile men from infertile groups. Furthermore, lactate, glutamate and cholesterol metabolites were deregulated and used to discriminate between ED and SA infertile men (25).

Zheng et al. applied GC-MS based plasma meta-bolomics profiles of infertile males with KYDS. They found 10 potential biomarkers including 1,5 Anhydroglucitol (The most important metabolite), α-Hydroxyvaleric acid, Galactose glucitol, Phenylalanine, Glutamic acid, L-Isoleucine, Phenylpropionic acid, N-acetylglycine, Ornithine and Lysine, and six metabolic pathways (Alanine, Aspartate and Glutamate metabolism, Arginine and Proline metabolism, Lysine degradation, Phenylalanine metabolism, Aminoacyl-tRNA biosynthesis, D-Glutamine and D-Glutamate metabolism) in plasma. These biomarkers can be used to distinguish infertile men with KYDS from fertile controls (26).

To the best of our knowledge, no further study has reported the use of plasma as a biological source to find potential biomarkers in infertile men.

### Human sperm metabolome:

To the best of our knowledge, there is only one human sperm meta-bolomics study which is metabolome mapping. Paiva et al. performed the only study of the human spermatozoa metabolome. They have identified 69 endogenous metabolites. They have combined NMR and GC-MS strategies to map the metabolome of human spermatozoa. The identified metabolites belonged to amino acids, carbohydrates, lipid super class, organic acids and aliphatic acrylic compounds. Additionally, they only did a small scale metabolomics profiling of human spermatozoa between asthenozoospermia (n=3) versus normospermic (n=3) men and they could not observe any statistically significant differences (27).

Since metabolomics is still under development in the male infertility field, it would be interested to see how the metabolome level can be changed in the human spermatozoa, such as asthenozoospermia patients.

## Discussion

Approximately 1,000 years ago the great Persian physician, Avicenna (980–1037), wrote in his famous book “The Canon of Medicine” (1025) about observation of the abnormal appetite, the collapse of sexual functions and the sweet taste and changes in urine color caused by diabetics. Nowadays, this is known to be caused by changing in the metabolites of urine at metabolome levels. Despite the fact that the word metabolome has been defined almost 20 years, only a handful of study can be found in the field of male infertility.

It is estimated that it will take almost 7 years to find a biomarker used in next high-throughput techniques. Metabolomics is a relative new field although targeted metabolomics (Inborn Errors of Metabolism) have the history of almost 100 years. Metabolomics study of infertile men is in the first steps of research. Few studies in which human seminal plasma, urine or plasma have been used as biological sources for finding the potential biomarkers of infertile men are available.

It has been successfully shown that with different metabolomics approaches, human seminal plasma is an excellent biological material source to categorize or find a potential biomarker of infertile men. At proteome levels, it has recently been shown that the human seminal plasma contains 2,168 non-redundant proteins (28). Assuming there is approximately the same number of metabolites in the human seminal plasma, it is far from identifying the metabolites in the human seminal plasma. A search in The Human Metabolome Database (Version 3.6) shows that seminal plasma is not a popular source of metabolomics researches for investigation of male infertility disorders yet. It can be found that there are only 9 human seminal plasma metabolites identified (29). The current metabolomics study of the human seminal plasma show that less than 40 metabolites are found from human seminal plasma (not published data).

To the best of our knowledge, one of the metabolomics techniques which have not been used to investigate the male reproductive system is LC-MS. This technique will increase the identification of some metabolites from human seminal plasma.

Although the application of the metabolomics approach is new in the male infertility, it is promising to find biomarkers and their etiology. However, this technique has not been applied to clinical works or diagnosis of infertile men. This is caused by the lack of metabolomics experts/trained persons in the field of the reproductive system.

## Conclusion

However, current researches on seminal plasma of infertile men reveal it would be wise to suggest introducing a new parameter to semen analysis in WHO laboratory manual for the examination and processing of human semen based on metabolomics technology, e.g. metabolic fingerprinting.

## References

[1] Hamada AJ Montgomery B Agarwal A Male infertility: a critical review of pharmacologic management. Expert Opin Pharmacother. 2012;13(17): 2511–31. 23121497 10.1517/14656566.2012.740011

[2] Kovac JR Pastuszak AW Lamb DJ The use of genomics, proteomics, and metabolomics in identifying biomarkers of male infertility. Fertil Steril. 2013; 99(4):998–1007. 23415969 10.1016/j.fertnstert.2013.01.111 PMC3652543

[3] Minai-Tehrani A Jafarzadeh N Gilany K Metabolomics: a state-of-the-art technology for better understanding of male infertility. Andrologia. 2016;48 (6):609–16. 26608970 10.1111/and.12496

[4] WHO WHO laboratory manual for the examination and processing of human semen. 5th ed Switzerland: World Health Organization; 2010 287 p.

[5] Strimbu K Tavel JA What are biomarkers? Curr Opin HIV AIDS. 2010;5(6):463–6. 20978388 10.1097/COH.0b013e32833ed177 PMC3078627

[6] Fiehn O Metabolomics--the link between genotypes and phenotypes. Plant Mol Biol. 2002;48(1–2):155–71. 11860207

[7] Goodacre R Metabolomics of a superorganism. J Nutr. 2007;137(1 Suppl):259S–66S. 17182837 10.1093/jn/137.1.259S

[8] Sue T Obolonkin V Griffiths H Villas-Boas SG An exometabolomics approach to monitoring microbial contamination in microalgal fermentation processes by using metabolic footprint analysis. Appl Environ Microbiol. 2011;77(21):7605–10. 21890679 10.1128/AEM.00469-11 PMC3209156

[9] Hamamah S Seguin F Barthelemy C Akoka S Le Pape A Lansac J 1H nuclear magnetic resonance studies of seminal plasma from fertile and infertile men. J Reprod Fertil. 1993;97(1):51–5. 8464025 10.1530/jrf.0.0970051

[10] Gilany K Pouracil RS Sadeghi MR Fourier transform infrared spectroscopy: a potential technique for noninvasive detection of spermatogenesis. Avicenna J Med Biotechnol. 2014;6(1):47–52. 24523955 PMC3895579

[11] da Silva BF Del Giudice PT Spaine DM Gozzo FC Turco EL Bertolla RP Metabolomics of male infertility: characterization of seminal plasma lipid fingerprints in men with spinal cord injury. Fertil Steril. 2011;96(3):S233.

[12] Jayaraman V Ghosh S Sengupta A Srivastava S Sonawat HM Narayan PK Identification of biochemical differences between different forms of male infertility by nuclear magnetic resonance (NMR) spectroscopy. J Assist Reprod Genet. 2014; 31(9):1195–204. 24965760 10.1007/s10815-014-0282-4 PMC4156941

[13] Gilany K Moazeni-Pourasil RS Jafarzadeh N Savadi-Shiraz E Metabolomics fingerprinting of the human seminal plasma of asthenozoospermic patients. Mol Reprod Dev. 2014;81(1):84–6. 24254375 10.1002/mrd.22284

[14] Zhang X Diao R Zhu X Li Z Cai Z Metabolic characterization of asthenozoospermia using non-targeted seminal plasma metabolomics. Clin Chim Acta. 2015;450:254–61. 26342261 10.1016/j.cca.2015.09.001

[15] Chen X Hu C Dai J Chen L Metabolomics analysis of seminal plasma in infertile males with kidney-yang deficiency: a preliminary study. Evid Based Complement Alternat Med. 2015;2015:892930. 25945117 10.1155/2015/892930 PMC4405216

[16] Gilany K Mani-Varnosfaderani A Minai-Tehrani A Mirzajani F Ghassempour A Sadeghi MR Untargeted metabolomic profiling of seminal plasma of non-obstructive azoospermia men: a non-invasive detection of spermatogenesis. Biomed Chromatogr. 2017;31(8):e3931. 10.1002/bmc.3931 28058728

[17] Gilany K Jafarzadeh N Mani-Varnosfaderani A Minai-Tehrani A Sadeghi MR Darbandi M Metabolic fingerprinting of seminal plasma from Non-obstructive azoospermia patients: positive versus negative sperm retrieval. J Reprod Infertil. 2018;19(2):109–14. 30009145 PMC6010822

[18] Zhang Z Zhang Y Liu C Zhao M Yang Y Wu H Serum metabolomic profiling identifies characterization of non-obstructive azoospermic men. Int J Mol Sci. 2017;18(2): pii: E238. 10.3390/ijms18020238 PMC5343775 28125052

[19] Deepinder F Chowdary HT Agarwal A Role of metabolomic analysis of biomarkers in the management of male infertility. Expert Rev Mol Diagn. 2007;7(4):351–8. 17620044 10.1586/14737159.7.4.351

[20] Sharma RK Agarwal A Role of reactive oxygen species in male infertility. Urology. 1996;48(6): 835–50. 8973665 10.1016/s0090-4295(96)00313-5

[21] Jafarzadeh N Mani-Varnosfaderani A Minai-Therani A Savadi-Shiraz E Sadeghi MR Gilany K Metabolomics fingerprinting of seminal plasma from unexplained infertile men: a need for novel diagnostic biomarkers. Mol Reprod Dev. 2015;82 (3):150. 25676838 10.1002/mrd.22457

[22] Qiao S Wu W Chen M Tang Q Xia Y Jia W Seminal plasma metabolomics approach for the diagnosis of unexplained male infertility. PloS One. 2017;12(8):e0181115. 28797078 10.1371/journal.pone.0181115 PMC5552325

[23] Zhang J Huang Z Chen M Xia Y Martin FL Hang W Urinary metabolome identifies signatures of oligozoospermic infertile men. Fertil Steril. 2014;102(1):44–53.e12. 24746742 10.1016/j.fertnstert.2014.03.033

[24] Zhang J Mu X Xia Y Martin FL Hang W Liu L Metabolomic analysis reveals a unique urinary pattern in normozoospermic infertile men. J Proteome Res. 2014;13(6):3088–99. 24796210 10.1021/pr5003142

[25] Zhou X Wang Y Yun Y Xia Z Lu H Luo J A potential tool for diagnosis of male infertility: Plasma metabolomics based on GC-MS. Talanta. 2016;147:82–9. 26592580 10.1016/j.talanta.2015.09.040

[26] Zheng P Wang Y Lu H Zhou X Tang T Fan R Plasma metabolomics analysis based on GCMS in infertile males with Kidney-Yang deficiency syndrome. Evid Based Complement Alternate Med. 2017;2017:6270195. 10.1155/2017/6270195 PMC5674502 29292399

[27] Paiva C Amaral A Rodriguez M Canyellas N Correig X Ballescà JL Identification of endogenous metabolites in human sperm cells using proton nuclear magnetic resonance (1H-NMR) spectroscopy and gas chromatography-mass spectrometry (GC-MS). Andrology. 2015;3(3): 496–505. 25854681 10.1111/andr.12027

[28] Gilany K Minai-Tehrani A Savadi-Shiraz E Rezadoost H Lakpour N Exploring the human seminal plasma proteome: an unexplored gold mine of bio-marker for male infertility and male reproduction disorder. J Reprod Infertil. 2015;16(2):61–71. 25927022 PMC4386088

[29] Wishart DS Jewison T Guo AC Wilson M Knox C Liu Y HMDB 3.0--the human metabolome database in 2013. Nucleic Acids Res. 2013; 41(Database issue):D801–7. 23161693 10.1093/nar/gks1065 PMC3531200

